# Increasing standardized ileal digestible arginine-to-lysine ratios improves growth performance and carcass characteristics in 10- to 31-d-old broilers

**DOI:** 10.1016/j.psj.2026.107015

**Published:** 2026-04-28

**Authors:** Inho Cho, Jisoo Tak, Changsu Kong

**Affiliations:** a Department of Animal Science and Biotechnology, Kyungpook National University, Sangju 37224, South Korea; b CJ Bio, CJ Cheiljedang Corp, Seoul 04560, South Korea; c Department of Animal Science, Kyungpook National University, Sangju 37224, South Korea; d Research Institute for Innovative Animal Science, Kyungpook National University, Sangju 37224, South Korea

**Keywords:** Arginine, Breast meat, Broiler, Growth performance, Requirement, Ideal ratio

## Abstract

This experiment was conducted to estimate the standardized ileal digestible (SID) arginine (Arg) to lysine (Lys) ratio requirements for broilers from 10 to 31 days of age. A total of 375 Ross 308 male broilers were assigned to 5 dietary treatments with 5 replicates of 15 birds using a randomized complete block design. Experimental diets set the SID Arg:Lys ratio of 85%, 95%, 105%, 115%, and 125%. Data were analyzed using the MIXED procedure, and Arg requirements were estimated using the NLIN procedure of SAS. During 10 to 21 days, a quadratic response in body weight gain (BWG) (P = 0.003) and a linear increase in gain-to-feed ratio (G:F) (P < 0.001) with increasing dietary SID Arg levels were observed. During 21 to 31 days, dietary SID Arg levels yielded a linear increase in BWG (P = 0.010) and a quadratic response for G:F (P = 0.026). Across 10 to 31 days, a quadratic response to dietary SID Arg levels was observed in BWG (P = 0.018) and G:F (P = 0.006). The dietary SID Arg levels increased linearly carcass yield (P = 0.005) and yielded quadratic responses for carcass weight (P = 0.029), breast meat weight (P = 0.003), and breast meat yield (P = 0.031). Estimated SID Arg:Lys ratio requirements, based on linear broken-line (LBL) and quadratic broken-line (QBL) models, ranged from 105.6% to 116.4% for BWG and 108.1% to 123.4% for G:F across the experimental periods. The LBL and QBL models were not significant for carcass yield. Dietary SID Arg levels linearly increased blood urea nitrogen (BUN; P = 0.027) and decreased mRNA expression of arginosuccinate synthase in liver (P = 0.025). In conclusion, the increasing dietary SID Arg:Lys ratio improved growth performance, carcass yield, and altered Arg metabolism in broilers. The optimal SID Arg:Lys requirements varied with response criteria used for estimation.

## Introduction

Arginine (Arg) is an indispensable amino acid (AA) for broilers because their endogenous Arg synthesis is not sufficient to meet the Arg requirement (Brugaletta et al., 2023; Ospina-Rojas et al., 2014). For effective growth, therefore, a sufficient supply of Arg is essential, as it plays a major role in immune function, growth, and metabolism (Al-Daraji and Salih, 2012; Hassan et al., 2021). However, excessive Arg supplementation is not effective, as it not only increases feed cost but also reduces Arg utilization due to AA imbalance and the antagonistic interaction between Arg and lysine (Lys) (Chamruspollert et al., 2002). Additionally, AA requirements have increased with genetic improvements in breed performance in broilers (Applegate and Angel, 2014; Dozier et al., 2008), therefore, continuous estimation of the optimal dietary Arg level is necessary to ensure accurate Arg provision.

The requirement for a full range of indispensable AA is almost impossible to estimate considering all factors with dose-response experiments, as the requirement is influenced by various dietary, environmental, and genetic factors (Khajali and Wideman, 2010; Mack et al., 1999). To address this problem, requirements for all indispensable AA are expressed as ratios to Lys, a concept commonly referred to as the ideal ratio. Furthermore, requirement estimation and diet formulation on a digestible AA basis are more appropriate for optimal growth performance than those based on total AA basis (Kumar et al., 2016), and the digestible AA in broilers are determined as standardized ileal digestible (SID) AA to avoid the effect of hindgut fermentation and to correct for basal endogenous losses of AA (Kong and Adeola, 2014). Therefore, expressing the Arg requirement of broilers as an ideal ratio based on SID AA is appropriate for accurate estimation.

To determine the AA requirements in broilers, growth performance has traditionally been used as the primary response (Sakomura et al., 2015). However, growth performance alone may not accurately reflect AA utilization for protein synthesis, as the response to dietary AA levels can vary with the specific response criteria (Nogueira et al., 2022; Sirathonpong et al., 2019). Carcass and breast meat yield are critical economic traits derived from protein accretion; therefore, establishing each Arg requirement aligned with carcass production allows for a more precise and efficient provision of Arg.

In addition, the Arg requirement can be influenced by environmental factors and immune status (Chamruspollert et al., 2004; Taghinejad-Roudbaneh et al., 2011). Recent research has demonstrated that providing Arg above recommended levels to broilers can improve growth performance and gut microbiota (Brugaletta et al., 2023), and additional Arg supplementation can maintain growth performance under heat stress conditions (Chamruspollert et al., 2004). These findings suggest that Arg requirements may be influenced by changes in Arg metabolism under various conditions, indicating that metabolic responses could be informative for requirement estimation. The evaluation of Arg metabolism at different Arg levels may be necessary to provide physiological insight into requirement estimation, as it may help to better interpret Arg requirements, and Arg metabolism can also be affected by different dietary Arg levels (Khajali and Wideman, 2010; Ruiz-Feria et al., 2001). Therefore, the current study aimed to estimate the optimal SID Arg:Lys ratio for growth performance and carcass characteristics and to evaluate the effect of Arg metabolism at different dietary Arg levels.

## Materials and methods

Experimental procedures were conducted in accordance with the Institutional Animal Care and Use Committee of Kyungpook National University, Republic of Korea (approval number: KNU 2024-0402).

### Animals and management

Post-hatched Ross 308 male broilers were assigned identification tags and housed in floor pens (100 × 100 × 60 cm) within an environmentally controlled room with continuous 24-hour lighting. Birds were fed with a broiler crumbled commercial diet containing 220 g/kg CP and 3,000 kcal/kg AMEn until day 10. Subsequently, a total of 375 birds were individually weighed and assigned to five dietary treatments, each comprising five replicates of 15 birds, in a randomized complete block design, using body weight (BW) as a blocking factor. The animal experiment was conducted during the grower phase from 10 to 21 days and the finisher phase from 21 to 31 days. All broilers had free access to both experimental diets in a mash form and water throughout the experimental period. The ambient temperature was maintained at 33°C for the first 4 days and then gradually decreased by 2°C per week until it reached 24°C. On day 21 and 31, individual BW and feed leftovers per pen were measured to calculate the body weight gain (BWG), feed intake, and gain to feed ratio (G:F), which were used to select birds corresponding to the median BW per pen for sampling.

### Dietary treatments

The experimental diets were corn–soybean meal–based and consisted of five diets varying SID Arg concentrations. All diets were formulated to set the ratio of SID Arg:Lys of 85%, 95%, 105%, 115%, and 125% for 10 to 21 days old and 21 to 31 days old broilers, while the SID Lys concentration was fixed across all experimental diets (Table 1, Table 2). The composition of all feed ingredients was the same across all experimental diets, except for corn starch, Arg, and alanine. To maintain the dietary CP level, alanine was added at the expense of cornstarch. The SID AA content in the experimental diets was calculated using the equations described by Kong and Adeola (2014). The dietary nutrients and metabolizable energy, except for Arg, were met or exceeded according to Aviagen (2022) recommendations to prevent any growth performance impairment due to nutrients other than Arg. The analyzed nutrient composition of the experimental diets was presented in Table 3, Table 4.

**Table 1:** Ingredient composition of experimental diets fed to grower broilers from 10 to 21 days.

Item, %	SID Arg:Lys 85%	SID Arg:Lys 95%	SID Arg:Lys 105%	SID Arg:Lys 115%	SID Arg:Lys 125%
Corn	62.10	62.10	62.10	62.10	62.10
Soybean meal	27.55	27.55	27.55	27.55	27.55
Soybean oil	2.20	2.20	2.20	2.20	2.20
Cornstarch	0.00	0.13	0.26	0.38	0.50
L-Arg	0.00	0.12	0.24	0.36	0.48
L-His-HCl	0.04	0.04	0.04	0.04	0.04
L-Ile	0.19	0.19	0.19	0.19	0.19
L-Lys-HCl	0.44	0.44	0.44	0.44	0.44
L-Met	0.43	0.43	0.43	0.43	0.43
L-Thr	0.21	0.21	0.21	0.21	0.21
L-Val	0.20	0.20	0.20	0.20	0.20
L-Ala	2.49	2.24	1.99	1.75	1.51
Limestone	1.05	1.05	1.05	1.05	1.05
Monocalcium phosphate	1.50	1.50	1.50	1.50	1.50
Sodium chloride	0.40	0.40	0.40	0.40	0.40
Vitamin premix	0.50	0.50	0.50	0.50	0.50
Mineral premix	0.50	0.50	0.50	0.50	0.50
Choline chloride	0.20	0.20	0.20	0.20	0.20
Total	100.00	100.00	100.00	100.00	100.00

Arg, arginine; Ala, alanine; His, histidine; Ile, isoleucine; Lys, lysine; Met, methionine; SID, standardized ileal digestible; Thr, threonine; Val, valine.

**Table 2:** Ingredient composition of experimental diets fed to finisher broilers from 21 to 31 days.

Item, %	SID Arg:Lys 85%	SID Arg:Lys 95%	SID Arg:Lys 105%	SID Arg:Lys 115%	SID Arg:Lys 125%
Corn	66.91	66.91	66.91	66.91	66.91
Soybean meal	23.81	23.81	23.81	23.81	23.81
Soybean oil	2.20	2.20	2.20	2.20	2.20
Cornstarch	-	0.12	0.24	0.35	0.46
L-Arg	-	0.11	0.22	0.33	0.44
L-His-HCl	0.03	0.03	0.03	0.03	0.03
L-Ile	0.19	0.19	0.19	0.19	0.19
L-Lys-HCl	0.41	0.41	0.41	0.41	0.41
L-Met	0.40	0.40	0.40	0.40	0.40
L-Thr	0.18	0.18	0.18	0.18	0.18
L-Val	0.18	0.18	0.18	0.18	0.18
L-Ala	1.94	1.71	1.48	1.26	1.04
Limestone	0.91	0.91	0.91	0.91	0.91
Monocalcium phosphate	1.24	1.24	1.24	1.24	1.24
Sodium chloride	0.40	0.40	0.40	0.40	0.40
Vitamin premix	0.50	0.50	0.50	0.50	0.50
Mineral premix	0.50	0.50	0.50	0.50	0.50
Choline chloride	0.20	0.20	0.20	0.20	0.20
Total	100.00	100.00	100.00	100.00	100.00

Arg, arginine; Ala, alanine; His, histidine; Ile, isoleucine; Lys, lysine; Met, methionine; SID, standardized ileal digestible; Thr, threonine; Val, valine.

**Table 3:** Nutrient concentrations of grower experimental diets.

Item, %	SID Arg:Lys 85%	SID Arg:Lys 95%	SID Arg:Lys 105%	SID Arg:Lys 115%	SID Arg:Lys 125%
Calculated value					
MEn, kcal/kg	3051	3052	3053	3054	3055
Crude protein, %	21.50	21.50	21.49	21.50	21.51
Ca, %	0.75	0.75	0.75	0.75	0.75
Non-phytate P, %	0.42	0.42	0.42	0.42	0.42
SID amino acids, g/kg^1^					
Arg	10.0	11.2	12.4	13.6	14.8
His	4.4	4.4	4.4	4.4	4.4
Ile	8.0	8.0	8.0	8.0	8.0
Leu	13.4	13.4	13.4	13.4	13.4
Lys	11.8	11.8	11.8	11.8	11.8
Met + Cys	9.2	9.2	9.2	9.2	9.2
Phe	7.9	7.9	7.9	7.9	7.9
Thr	7.9	7.9	7.9	7.9	7.9
Trp	2.0	2.0	2.0	2.0	2.0
Val	9.1	9.1	9.1	9.1	9.1
Analyzed value, %					
Crude protein	21.9	21.7	21.5	21.5	21.7
Arg	11.2	12.3	13.0	14.1	15.3
His	4.8	4.7	4.8	4.6	5.1
Ile	9.1	9.3	9.1	8.8	9.2
Leu	16.0	16.1	15.8	15.7	16.0
Lys	13.2	13.4	13.1	13.3	13.1
Met	4.5	3.2	4.7	3.8	4.6
Cys	0.0	0.0	0.0	0.0	0.0
Phe	9.8	9.8	9.6	9.7	9.8
Thr	9.4	9.9	9.4	9.5	9.4
Trp	1.0	1.1	1.1	1.0	1.0
Val	10.2	10.3	9.9	10.3	10.5

Arg, arginine; Ala, alanine; Cys, cysteine; His, histidine; Ile, isoleucine; Leu, leucine; Lys, lysine; Met, methionine; MEn = nitrogen-corrected metabolizable energy; P, phosphorus; Phe, phenylalanine; SID, standardized ileal digestible; Thr, threonine; Trp, tryptophan Val, valine.

1 The ideal amino acid ratios of treatments, except for Arg, are presented below: His (37%), Ile (68%), Leu (110%), Met + Cys (78%), Phe (63%), Thr (67%), Trp (16%), Val (77%).

**Table 4:** Nutrient concentrations of finisher experimental diets.

Item, %	SID Arg:Lys 85%	SID Arg:Lys 95%	SID Arg:Lys 105%	SID Arg:Lys 115%	SID Arg:Lys 125%
Calculated value					
MEn, kcal/kg	3106	3107	3108	3109	3055
Crude protein, %	19.47	19.46	19.46	19.46	21.51
Ca, %	0.65	0.65	0.65	0.65	0.75
Non-phytate P, %	0.36	0.36	0.36	0.36	0.42
SID amino acids, g/kg^1^					
Arg	10.0	11.2	12.4	13.6	10.0
His	4.4	4.4	4.4	4.4	4.4
Ile	8.0	8.0	8.0	8.0	8.0
Leu	13.4	13.4	13.4	13.4	13.4
Lys	11.8	11.8	11.8	11.8	11.8
Met + Cys	9.2	9.2	9.2	9.2	9.2
Phe	7.9	7.9	7.9	7.9	7.9
Thr	7.9	7.9	7.9	7.9	7.9
Trp	2.0	2.0	2.0	2.0	2.0
Val	9.1	9.1	9.1	9.1	9.1
Analyzed value, %					
Crude protein	20.0	20.3	19.8	19.6	19.8
Cit	0.0	0.0	0.0	0.0	0.0
Arg	10.0	10.7	11.6	12.9	13.4
His	4.4	4.3	4.4	4.1	4.2
Ile	8.7	8.4	8.3	8.6	8.5
Leu	15.0	14.8	14.6	14.8	14.6
Lys	12.1	11.8	11.7	11.8	11.6
Met	4.1	4.3	5.1	3.9	3.5
Cys	0.0	0.0	0.0	0.0	0.0
Phe	9.0	8.9	8.8	8.9	8.8
Thr	8.7	8.4	8.4	8.6	8.4
Trp	1.1	1.2	1.1	1.1	1.1
Val	9.5	9.5	9.5	9.5	9.5

Arg, arginine; Ala, alanine; Cys, cysteine; His, histidine; Ile, isoleucine; Leu, leucine; Lys, lysine; Met, methionine; MEn = nitrogen-corrected metabolizable energy; P, phosphorus; Phe, phenylalanine; SID, standardized ileal digestible; Thr, threonine; Trp, tryptophan Val, valine.

1 The ideal amino acid ratios of treatments, except for Arg, are presented below: His (37%), Ile (69%), Leu (110%), Met + Cys (80%), Phe (63%), Thr (67%), Trp (16%), Val (78%).

### Carcass, breast meat, blood, and organ sampling

On day 32, a bird in each pen was selected based on the median BW for sampling and euthanized by CO_2_ asphyxiation. After euthanasia, the blood samples were collected from the jugular vein into the vacutainer EDTA tube. Abdominal fat, entire liver, and kidney samples were then excised, collected, and weighed, followed by carcass sampling for weight measurement and subsequent collection of the entire bilateral breast meat samples. Carcass weight was recorded as the weight of the bird after removing the feathers, head, feet, viscera, abdominal fat (Leeson and Summers, 1980). The relative weights of these samples were expressed as a percentage of the BW. The collected kidney and liver tissue samples were immediately frozen using liquid nitrogen and stored at - 80°C for later analysis.

### Breast meat characteristics

Immediately upon arrival in the lab, the complete Commission Internationale de l'Eclairage (CIE) system color profile of lightness (L*), redness (a*), and yellowness (b*) was measured on individual breast meat samples. Breast meat samples from both sides were processed separately, and the breast meat sample was cut in half horizontally, and the meat color of the cross-sectional area was measured using a meat colorimeter (Konica Minolta Inc., CR-400, Chiyoda, Tokyo, Japan) in duplicate. Afterward, the breast meat samples were cut into about 10 g and immediately vacuum-packed in a plastic bag to store at 4°C for 24 hours. After refrigeration, the sample was wiped with absorbent paper and weighed again. The difference in weight corresponded to the drip loss and was expressed as the percentage of the initial weight. Approximately 2.5 g of breast meat was homogenized with distilled water at three times its weight for 1 min. The homogenate was centrifuged at 3,000 g for 10 min, filtered through Whatman No. 4 filter paper, and the filtrate was measured with a pH meter (OHAUS Corp., ST3100-F, Parsippany, NJ).

### Plasma metabolite analysis

The collected blood samples were centrifuged at 2,000 g for 10 min at 4°C to obtain plasma and stored at - 20°C for later analysis. The plasma samples were used to measure the concentration of blood urea nitrogen (BUN) using an automated chemistry analyzer (Fujifilm Corp., Fuji Dri-chem 3500i, Minato, Tokyo, Japan).

### Nitric oxide and enzymes associated with arginine metabolism activity

Liver and kidney samples were used for determination of nitric oxide (NO) concentration using commercial nitric oxide assay kits (Abcam, ab272517, Cambridge, Cambridgeshire, United Kingdom). In this method, total nitrate and nitrite (NO₃⁻/NO₂⁻) derived from NO were first reduced to nitrite, and the resulting nitrite was then quantified by a colorimetric method according to the manufacturer’s instructions. Enzymes involved in arginine metabolism including total nitric oxide synthase (tNOS), were measured using commercial test kit (Abcam, ab211084, Cambridge, Cambridgeshire, United Kingdom) which is fluorometric method and arginase using chicken ELISA kit (Antibodies, ABIN1051107, Cambridge, Cambridgeshire, United Kingdom). The reaction absorbance for NO and tNOS was determined at 540 nm using a microplate reader (BioTek Inc., EPOCH, Winooski, VT) while arginase was read at 450 nm and the standard curve was used to compute the sample concentration.

### Gene expression

The total RNA was extracted from the kidney and liver samples using the TRizolTM Reagent (Thermo Fisher Scientific, Waltham, MA). Subsequently, samples were homogenized using a homogenizer (Bertin Technologies, Precellys®24, Montigny-le-Bretonneux, Yvelines, France). The isolated RNA was used for one-step RT-qPCR using AccuPower® GreenStar™ RT-qPCR Master Mix (Bioneer, Daejeon, Chungcheong Province, Republic of Korea) with specific primers for argininosuccinate synthase (ASS) and argininosuccinate lyase (ASL) (Table 5). For investigation of mRNA expression level, qRT-PCR was performed using Roter-Gene Q instrument (Qiagen, Hilden, North Rhine-Westphalia, Germany) with the following reaction conditions: initial reverse transcription 50°C for 15 min, initial denaturation 95°C for 5 min, followed by 40 cycles of denaturation at 95°C for 15 sec and annealing/extension at 60°C for 30 sec. The target gene expression levels were normalized against β-actin as the housekeeping gene, while the SID Arg:Lys 85% diet was used as the calibrator. The Ct values of the reference genes were adjusted to remove ΔCt bias by using a regression method that accounts for the difference in PCR amplification efficiency between target and reference genes, as described by Cui et al. (2015).

**Table 5:** Primer sequences (5′−3′) used for house-keeping genes and target genes for real-time PCR assays.

Item		5′−3′ primer sequence
Arginosuccinate synthase	Forward	AGAACCTCATGCACATCAGC
	Reverse	TTGGGTTGCAGGTCTTTGTG
Arginosuccinate lyase	Forward	GGGAGTCTTTGTGGTGAAACAA
	Reverse	TGCAGCAGGTGAGTGGAAAT
β-actin	Forward	TTGTCCACCGCAAATGCTTC
	Reverse	AAAGCCATGCCAATCTCGTC

### Chemical analysis

The experimental diets were ground using a mill grinder (CT 293 Cyclotec, Foss Ltd., Denmark) for chemical analysis. The experimental diets were analyzed for AA (method 994.12; method 999.13; method 988.15; method 985.28), CP (method 968.08), calcium, and phosphorus (method 965.17) contents as described in AOAC (2016).

### Statistical analysis

Growth performance, carcass characteristics, breast meat quality, and Arg metabolism–related data were analyzed using the MIXED procedure of the SAS software (SAS 9.4, SAS Inst. Inc., Cary, NC, USA). Dietary treatment was regarded as a fixed variable, while the replicate was considered as a random variable. The Ct values of the reference gene were subjected to linear regression against those of the target gene, and the reference gene values were adjusted with the estimated regression coefficient to remove bias. The corrected ΔCt values were analyzed using the same MIXED procedure as that applied to the previous dataset. The experimental unit was a pen. Linear and quadratic effects of dietary treatment were identified using orthogonal polynomial contrasts. The SID Arg requirements were estimated for linear broken-line (LBL) and quadratic broken-line (QBL) models using the NLIN procedure of SAS based on the study by Robbins et al. (2006). Statistical significance and tendency were set at P < 0.05 and 0.05 ≤ P < 0.1, respectively.

## Results

### Growth performance

Table 6 presents data on growth performance of broilers from 10 to 21 days, 21 to 31 days, and 10 to 31 days. During 10 to 21 days, a quadratic increase in BWG was observed in broilers fed experimental diets with increasing SID Arg levels (P = 0.003). The dietary SID Arg level increased linearly the G:F (P < 0.001). During 21 to 31 days, BWG increased linearly with increasing dietary SID Arg level (P = 0.010), and G:F quadratically responded to increasing dietary SID Arg level (P = 0.026). During 10 to 31 days, quadratic responses in both BWG (P = 0.018) and G:F (P = 0.006) of broilers with increasing dietary SID Arg level were observed.

**Table 6:** Growth performance of male broilers fed experimental diets.

Item	Dietary treatments					SEM	P-value	
	SID Arg/Lys 85%	SID Arg/Lys 95%	SID Arg/Lys 105%	SID Arg/Lys 115%	SID Arg/Lys 125%		Linear	Quadratic
10-21 days								
BWG, g	538	567	592	609	591	22.0	< 0.001	0.003
FI, g	757	775	793	802	780	30.7	0.057	0.057
G:F g/kg	712	731	747	761	758	6.3	< 0.001	0.059
21-31 days								
BWG, g	754	779	807	815	801	19.3	0.010	0.083
FI, g	1,234	1,231	1,249	1,246	1,230	30.1	0.856	0.373
G:F g/kg	612	633	646	654	651	6.0	< 0.001	0.026
10-31 days								
BWG, g	1,292	1,345	1,399	1,424	1,392	39.9	< 0.001	0.018
FI, g	1,990	2,006	2,041	2,047	2,010	59.8	0.269	0.131
G:F g/kg	650	671	685	696	692	4.7	< 0.001	0.006

Arg, arginine; BWG, body weight gain; FI, feed intake; G:F, gain-to-feed ratio; Lys, lysine; SEM, standard error of the mean; SID, standardized ileal digestible.

**Fig. 1:**
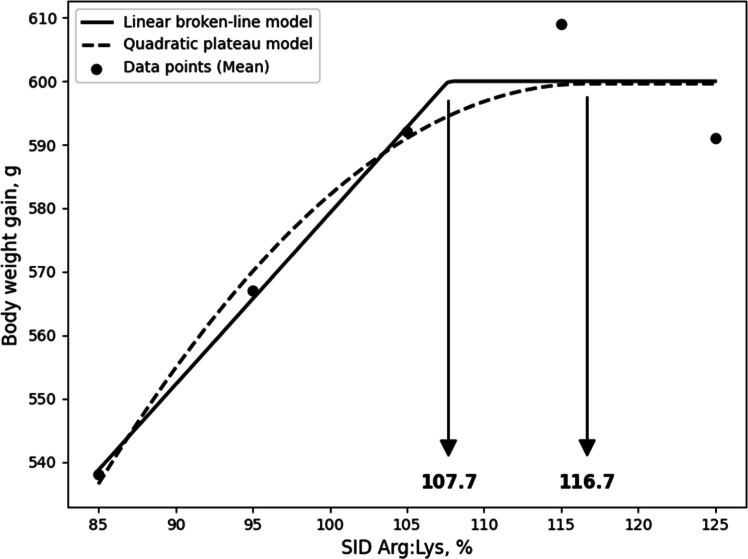
Fitted linear broken (solid) and quadratic (dot) lines of body weight gain (g) for 10 to 21-day-old broilers as function of ideal ratio of the standardized ileal digestible (SID) Arg to SID Lys in the diet. Data points express means of 5 replicates per treatment. The linear broken-line model shows that the ideal ratio of SID Arg to SID Lys is 107.7% (Y = 600 – 2.7[107.7 – X] [X < 107.7]; P = 0.054; R^2^ = 0.946). The quadratic line model shows that the ideal ratio of SID Arg to SID Lys is 116.7% (Y = 599.6 – 0.0668[116.7 – X] [116.7 – X] [X < 116.7]; P = 0.059; R^2^ = 0.941). Arg, arginine; Lys, lysine; SID, standardized ileal digestible.

**Fig. 2:**
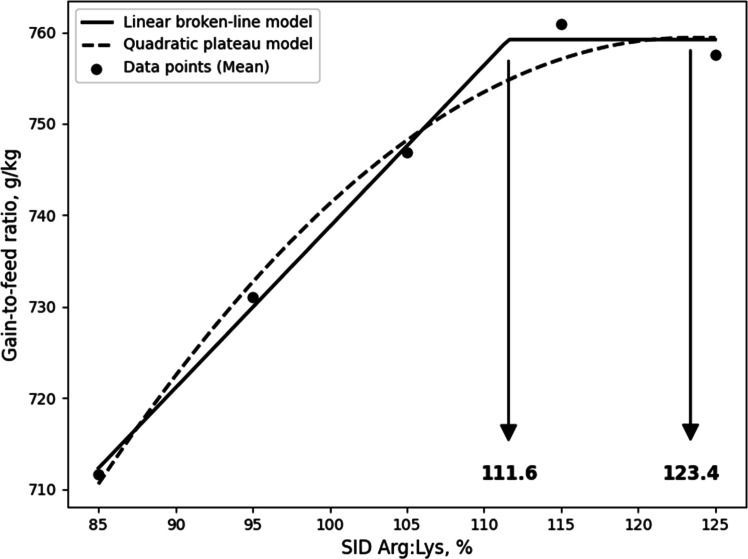
Fitted linear broken (solid) and quadratic (dot) lines of gain-to-feed ratio (g/kg) for 10 to 21-day-old broilers as function of the ideal ratio of standardized ileal digestible (SID) Arg to SID Lys in the diet. Data points express means of 5 replicates per treatment. The linear broken-line model shows that the ideal ratio of SID Arg to SID Lys is 111.6% (Y = 759.2 – 1.762[111.6 – X] [X < 111.6]; P = 0.005; R^2^ = 0.996). The quadratic line model shows that the ideal ratio of SID Arg to SID Lys is 123.4% (Y = 759.4 – 0.0331[123.4 – X] [123.4 – X] [X < 123.4]; P = 0.014; R^2^ = 0.986). Arg, arginine; Lys, lysine; SID, standardized ileal digestible.

**Fig. 3:**
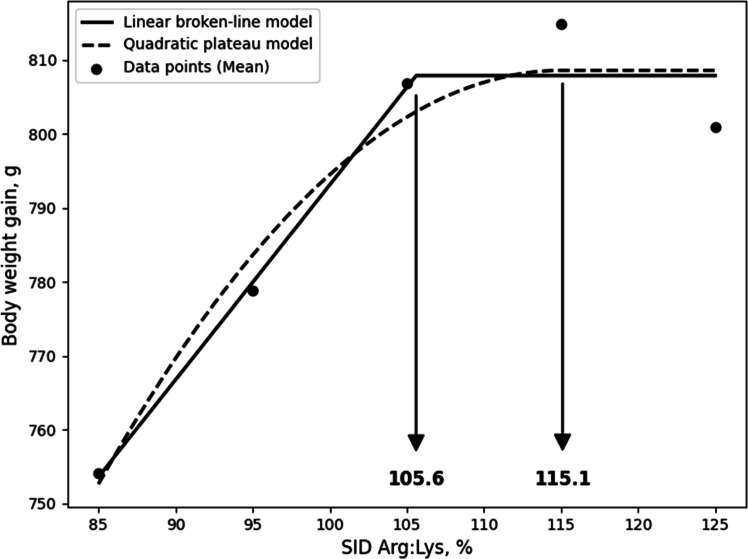
Fitted linear broken (solid) and quadratic (dot) lines of body weight gain (g) for 21 to 31-day-old broilers as function of the ideal ratio of standardized ileal digestible (SID) Arg to SID Lys in the diet. Data points express means of 5 replicates per treatment. The linear broken-line model shows that the ideal ratio of SID Arg to SID Lys is 105.6% (Y = 807.9 – 2.637[105.6 – X] [X < 105.6]; P = 0.041; R^2^ = 0.959). The quadratic line model shows that the ideal ratio of SID Arg to SID Lys is 115.1% (Y = 808.6 – 0.0618[115.1 – X] [115.1 – X] [X < 115.1]; P = 0.060; R^2^ = 0.940). Arg, arginine; Lys, lysine; SID, standardized ileal digestible.

**Fig. 4:**
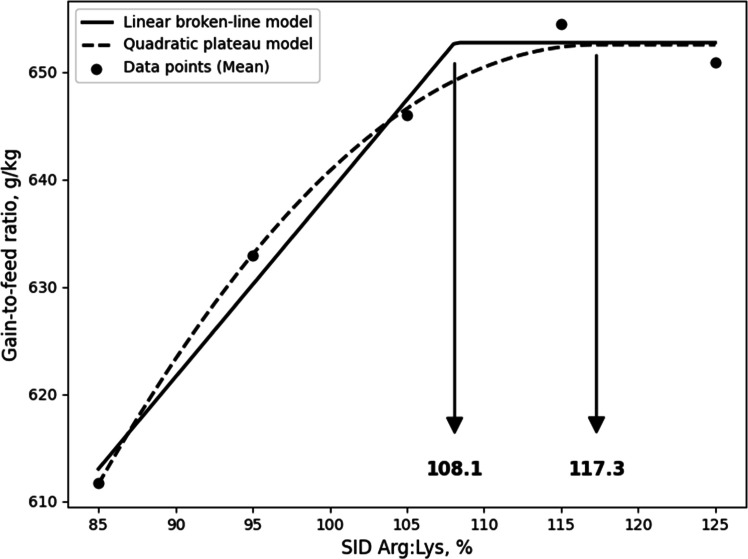
Fitted linear broken (solid) and quadratic (dot) lines of gain-to-feed ratio (g/kg) for 21 to 31-day-old broilers as function of the ideal ratio of standardized ileal digestible (SID) Arg to SID Lys in the diet. Data points express means of 5 replicates per treatment. The linear broken-line model shows that the ideal ratio of SID Arg to SID Lys is 108.1% (Y = 652.7 – 1.718[108.1 – X] [X < 108.1]; P = 0.014; R^2^ = 0.986). The quadratic line model shows that the ideal ratio of SID Arg to SID Lys is 117.3% (Y = 652.5 – 0.0392[117.3 – X] [117.3 – X] [X < 117.3]; P = 0.006; R^2^ = 0.994). Arg, arginine; Lys, lysine; SID, standardized ileal digestible.

**Fig. 5:**
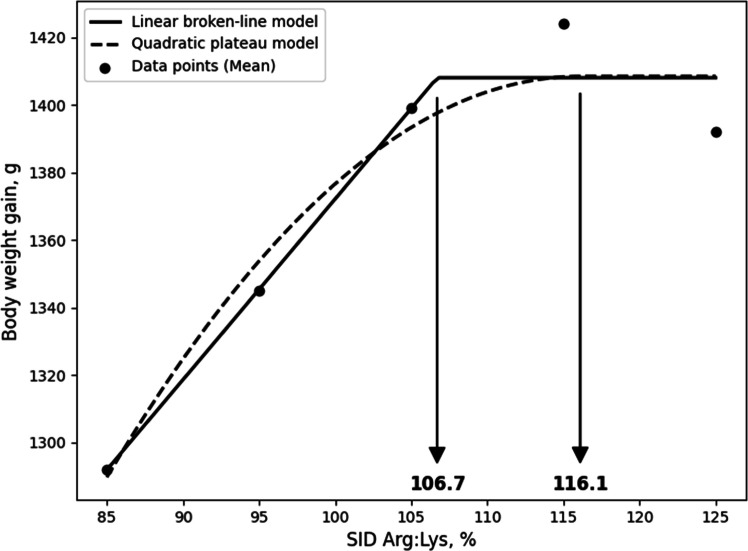
Fitted linear broken (solid) and quadratic (dot) lines of body weight gain (g) for 10 to 31-day-old broilers as function of the ideal ratio of standardized ileal digestible (SID) Arg to SID Lys in the diet. Data points express means of 5 replicates per treatment. The linear broken-line model shows that the ideal ratio of SID Arg to SID Lys is 106.7% (Y = 1408 – 5.35[106.7 – X] [X < 106.7]; P = 0.047; R^2^ = 0.953). The quadratic line model shows that the ideal ratio of SID Arg to SID Lys is 116.1% (Y = 1408.4 – 0.1227[116.1 – X] [116.1 – X] [X < 116.1]; P = 0.049; R^2^ = 0.240). Arg, arginine; Lys, lysine; SID, standardized ileal digestible.

**Fig. 6:**
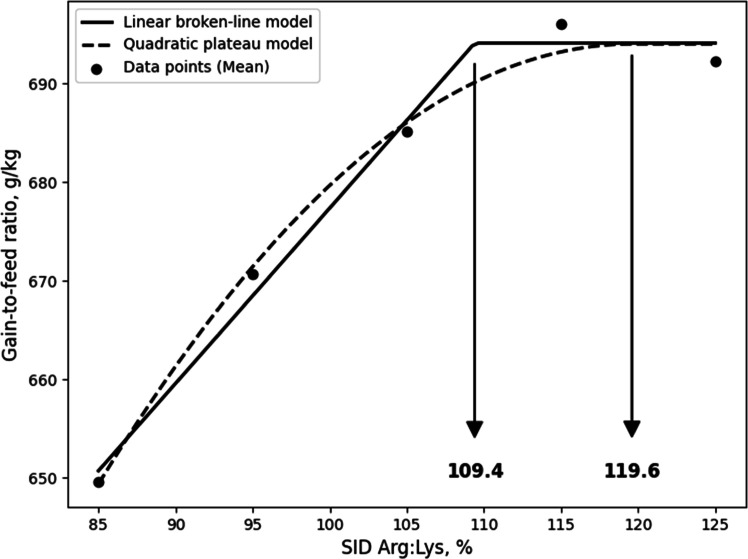
Fitted linear broken (solid) and quadratic (dot) lines of gain-to-feed ratio (g/kg) for 10 to 31-day-old broilers as function of the ideal ratio of standardized ileal digestible (SID) Arg to SID Lys in the diet. Data points express means of 5 replicates per treatment. The linear broken-line model shows that the ideal ratio of SID Arg to SID Lys is 109.4% (Y = 694.1 – 1.779[109.4 – X] [X < 109.4]; P = 0.010; R^2^ = 0.990). The quadratic line model shows that the ideal ratio of SID Arg to SID Lys is 119.6% (Y = 694 – 0.0373[119.6 – X] [119.6 – X] [X < 119.6]; P = 0.009; R^2^ = 0.991). Arg, arginine; Lys, lysine; SID, standardized ileal digestible.

Although the LBL and QBL models were not significant, they showed a tendency toward significance and relatively high R^2^ values, and, the estimated ideal ratios of SID Arg to SID Lys for BWG in 10- to 21-day-old broilers based on LBL and QBL were 107.7% (P = 0.054, R^2^ = 0.946) and 116.7% (P = 0.059, R^2^ = 0.941), respectively. (Fig. 1). For G:F, the estimated ratios based on the LBL and QBL models were 111.6% (P = 0.005, R^2^ = 0.996) and 123.4% (P = 0.014, R^2^ = 0.986), respectively (Fig. 2). In 21 to 31-day old broilers, the estimated SID Arg to SID Lys ratios for BWG were 105.6% (P = 0.041, R^2^ = 0.959) and 115.1% (P = 0.060, R^2^ = 0.940) using the LBL and QBL models, respectively (Fig. 3). The QBL model was not significant but showed a high R^2^ value. The estimated ratios of SID Arg to SID Lys for G:F in 21 to 31-day-old broilers were 108.1% (P = 0.014, R^2^ = 0.986) and 117.3% (P = 0.006, R^2^ = 0.994), respectively (Fig. 4). Finally, the estimated SID Arg to SID Lys ratios for BWG for 10 to 31-day-old broilers were 106.7% (P = 0.047, R^2^ = 0.953) and 116.1% (P = 0.049, R^2^ = 0.240) based on the LBL and QBL models, whereas the corresponding ratios for G:F were 109.4% (P = 0.010, R^2^ = 0.990) and 119.6% (P = 0.009, R^2^ = 0.991), respectively (Fig. 5, Fig. 6).

### Carcass characteristics

The carcass characteristics result is presented in Table 7. The dietary SID Arg levels result in a quadratic response for carcass weight (P = 0.029) and increased linearly carcass yield of broilers (P = 0.005). Breast meat weight and yield showed a quadratic response to dietary SID Arg levels (P = 0.003; P = 0.031). The percentage of liver decreased linearly with increasing the dietary SID Arg levels (P = 0.019), while the dietary SID Arg levels did not affect the percentage of kidney and abdominal fat.

**Table 7:** Carcass characteristics and percentage of organs in 32-day-old broiler fed experimental diets.

Item	Dietary treatments					SEM	P-value	
	SID Arg/Lys 85%	SID Arg/Lys 95%	SID Arg/Lys 105%	SID Arg/Lys 115%	SID Arg/Lys 125%		Linear	Quadratic
Median BW, g	1,722	1,737	1,848	1,846	1,769	-	-	-
Carcass weight, g	847	853	932	931	917	27.1	< 0.001	0.029
Carcass yield, %	49.3	49.1	50.4	50.5	51.9	0.72	0.005	0.403
Breast meat weight, g	151	165	183	193	177	8.1	< 0.001	0.003
Breast meat yield, %	8.8	9.5	9.9	10.4	10.0	0.28	< 0.001	0.031
Liver, %	2.5	2.5	2.3	2.2	2.2	0.11	0.019	0.827
Kidney, %	0.6	0.6	0.6	0.6	0.6	0.03	0.446	0.254
Abdominal fat, %	1.4	1.0	1.3	1.2	1.1	0.12	0.410	0.474

Arg, arginine; BW, body weight; Lys, lysine; SEM, standard error of the mean; SID, standardized ileal digestible.

The LBL and QBL models for carcass weight, carcass yield, breast meat weight, and breast meat yield were generally not significant (Table 8). Although the LBL model for carcass yield was statistically significant (P = 0.022, R^2^ = 0.866), it did not yield a meaningful breakpoint and was therefore not considered applicable. In contrast, for breast meat yield, the non-linear regression models showed a tendency toward significance with relatively high R^2^ values and the estimated breakpoints from the LBL and QBL models were 110.3% (P = 0.065, R^2^ = 0.935) and 118.4% (P = 0.070, R^2^ = 0.930), respectively.

**Table 8:** Linear broken-line and quadratic broken-line analysis of carcass and breast meat production responses of 32-day-old broilers to varying levels of dietary standardized ileal digestible arginine levels.

Item	Carcass		Breast meat	
	weight, g	yield, %	weight, g	yield, %
Linear broken-line model				
Slope (L)	NS	51.6	193	10.2
Asymptote (V)	NS	−0.07	−0.80	−0.05
Breakpoint (R)	NS	125.7	128.3	110.3
R^2^	NS	0.866	0.603	0.935
P-value	NS	0.022	0.1223	0.065
Quadratic broken-line model				
Slope (L)	926	NS	185	10.2
Asymptote (V)	−0.08	NS	−0.04	0.001
Breakpoint (R)	119.3	NS	116.1	118.4
R^2^	0.794	NS	0.858	0.930
P-value	0.206	NS	0.142	0.070

NS, not significant.

### Breast meat quality

The breast meat quality result is presented in Table 9. The dietary SID Arg levels decreased linearly pH of breast meat (P < 0.001) while it increased linearly drip loss of breast meat (P = 0.048). Additionally, the dietary SID Arg levels increased linearly L* (P < 0.001) and b* (P = 0.002) of breast meat, however it decreased linearly a* of breast meat (P = 0.002).

**Table 9:** Breast meat quality and meat color of broiler fed experimental diets.

Item			Meat color		
	pH	Drip loss, %	L*	a*	b*
Dietary treatments					
SID Arg/Lys 85%	6.17	9.42	53.7	3.0	7.3
SID Arg/Lys 95%	6.31	10.19	52.6	2.7	6.8
SID Arg/Lys 105%	6.05	11.83	54.9	2.3	7.7
SID Arg/Lys 115%	5.94	13.16	57.0	2.0	8.8
SID Arg/Lys 125%	5.89	11.28	58.2	1.7	9.0
SEM	0.062	1.381	1.03	0.34	0.56
P-value					
Linear	< 0.001	0.048	< 0.001	0.002	0.002
Quadratic	0.346	0.150	0.215	0.989	0.350

a*, redness; Arg, arginine; b*, yellowness; L*, lightness; Lys, lysine; SEM, standard error of the mean; SID, standardized ileal digestible.

### Blood metabolite, nitric oxide concentration and enzyme activity associated with arginine metabolism

The results of BUN concentration, NO concentration, NOS activity, and arginase activity are presented in Table 10. The dietary SID Arg levels increased linearly the concentration of BUN (P = 0.027). The concentration of NO and activity of NOS and arginase in liver and kidney was not affected by dietary SID Arg levels.

**Table 10:** Plasma metabolite, nitric oxide concentration and enzyme activity associated with arginine metabolism.

Item	BUN, pg/dL	Liver			Kidney		
		NO, μM	NOS, U	Arginase pg/mL	NO, μM	NOS, U	Arginase pg/mL
Dietary treatments							
SID Arg/Lys 85%	7.40	29.7	0.064	291.0	4.0	0.014	111.4
SID Arg/Lys 95%	7.46	35.9	0.108	289.9	11.4	0.032	104.3
SID Arg/Lys 105%	7.92	24.6	0.034	290.8	3.4	0.028	108.9
SID Arg/Lys 115%	8.00	29.6	0.049	304.4	16.1	0.021	107.5
SID Arg/Lys 125%	8.22	35.4	0.113	295.8	13.1	0.021	105.6
SEM	0.288	3.78	0.0324	9.16	5.62	0.0128	4.80
P-value							
Linear	0.027	0.663	0.687	0.429	0.199	0.923	0.471
Quadratic	0.956	0.264	0.243	0.953	0.988	0.457	0.744

Arg, arginine; BUN, blood urea nitrogen; Lys, lysine; NO, nitric oxide; NOS, nitric oxide synthase; SEM, standard error of the mean; SID, standardized ileal digestible.

### mRNA expression of arginine synthesis enzymes in liver and kidney

The results of mRNA expression of ASS and ASL in liver and kidney are presented in Table 11. The dietary SID Arg levels decreased linearly mRNA expression of ASS while it did not affect the expression of ASL in liver (P = 0.025). The mRNA expression of ASS and ASL was not affected by dietary SID Arg levels.

**Table 11:** mRNA expression of enzymes associated with arginine synthesis.

Item	Liver		Kidney	
	ASS (ΔCT)	ASL (ΔCT)	ASS (ΔCT)	ASL (ΔCT)
Dietary treatments				
SID Arg/Lys 85%	4.18	9.08	0.81	4.70
SID Arg/Lys 95%	3.94	9.48	0.89	5.04
SID Arg/Lys 105%	3.92	9.53	1.13	4.99
SID Arg/Lys 115%	2.50	7.51	0.94	4.62
SID Arg/Lys 125%	3.26	9.09	0.75	4.68
SEM	0.429	0.965	0.150	0.163
P-value				
Linear	0.025	0.499	0.845	0.368
Quadratic	0.710	0.929	0.080	0.176

Arg, arginine; ASL, arginosuccinate lyase; ASS, arginosuccinate synthase; Lys, lysine; SEM, standard error of the mean; SID, standardized ileal digestible.

## Discussion

In the current study, both BWG and G:F increased linearly or responded quadratically as SID Arg:Lys ratio increased, irrespective of the experimental period, which is consistent with previous studies (Castro et al., 2020; Oliveira et al., 2022). These responses exhibited biologically curvilinear patterns, indicating that the application of a broke-line model was appropriate. This may be attributed to the alleviation of Arg deficiency through higher dietary Arg levels. The SID Arg:Lys ratios in the experimental diets spanned 85% to 125%, encompassing the recommended level of approximately 107% (Aviagen, 2022; Rostagno et al., 2017). Therefore, the observed responses are consistent with a typical biological response, supporting the validity of the experimental design and subsequent requirement estimation.

The asymptote values at breakpoint were comparable between LBL model and QBL, whereas previous studies reported that the asymptote values in QBL model are numerically greater than those of LBL model (An and Kong, 2024; An et al., 2023). If the data response is clearly quadratic as the requirement is approached, the LBL model is not appropriate, and it will underestimate the breakpoint (Robbins et al., 2006). However, the data response in the present study was clearly linear as the requirement was approached, therefore, the underestimation of breakpoint in LBL model is alleviated, resulting in increase of asymptote value. Accordingly, the AA requirements in the present study was defined as the breakpoint, because the asymptote values derived from linear and quadratic models were comparable, unlike in previous studies that suggested AA requirements based on an arbitrarily defined target of 95% of the upper asymptote value of the quadratic line (An and Kong, 2024; An et al., 2023).

In the current study, although not statistically significant, some models showed a tendency toward significance with high R² values, indicating a good fit to the data. In addition, the estimated breakpoints were consistent with the other requirements for growth performance; therefore, these breakpoint estimates may still provide useful guidance for evaluating Arg requirements, despite the lack of statistical significance. As a result of the LBL model, it was calculated that broilers optimized their BWG during 10 to 21 days, 21 to 31 days, and 10 to 31 days at SID Arg:Lys ratios of 107.7%, 105.6%, and 106.7%, respectively, while the SID Arg:Lys ratios based on G:F were 111.6%, 108.1%, and 109.4%, respectively. The estimated SID Arg:Lys ratios for BWG align with current Arg recommendations for broilers. Notably, the requirements for G:F were numerically greater than those for BWG, consistent with previous studies reporting that the Arg requirements for G:F were greater than those of BWG (Corzo, 2012; Corzo et al., 2021; Jahanian, 2009). Similarly, the QBL model estimated SID Arg:Lys ratios for BWG at 116.7%, 115.1%, and 116.1% for the respective periods, while those for G:F were 123.4% 117.3%, and 119.6%. The breakpoint values of QBL model for growth performance exhibited a similar trend to the LBL model but were slightly greater than those of LBL model. This is because the LBL model estimate is more representative of the average requirement of the population, whereas the QBL model estimate is intended to satisfy a higher percentage of the population (Robbins et al., 2006). Therefore, the SID Arg:Lys ratio derived from BWG using the LBL model may be appropriate for meeting average growth requirements, whereas higher estimates based on G:F or QBL models may be more suitable when optimizing feed efficiency or maximizing production performance.

Additionally, the estimated SID Arg:Lys requirements for BWG remained relatively constant across the experimental periods. However, subtle variations were observed in G:F requirements, particularly when estimated via the QBL model, with a tendency to decrease as birds aged. The discrepancy between BWG and G:F observed in this study suggests that G:F may be a more sensitive indicator of age-related changes in AA metabolism than BWG. These age-dependent shifts, although minor in this study, align with previous findings indicating that the ideal AA profile may shift as birds mature. For instance, Emmert and Baker (1997) reported that the ideal Arg:Lys ratio increases from 105% (0 to 21 days) to 108% (21 to 42 days) to account for increasing maintenance requirements relative to tissue accretion. Baker (2009) further supported this concept, explaining that as birds age, the increasing demand for maintenance can shift the optimal ratio of several AA relative to Lys. However, the age-related changes in the estimated SID Arg:Lys ratio appear to be conflicting between the present study and previous reports, and the underlying mechanisms remain unclear. Given these observations, further research is warranted to elucidate these mechanisms and to evaluate the impact of both age and response criteria on estimating the ideal ratio.

Increase in dietary SID Arg level improved carcass weight and yield as it improved growth performance, except for the percentage of organs and abdominal fat, indicating that carcass weight increased with increasing dietary SID Arg levels more than organ and abdominal fat weight. The quadratic responses of carcass weight, breast meat weight, and yield of breast meat may reflect the rapid muscle accretion that occurs once limitations on protein synthesis are alleviated by sufficient Arg supply, and beyond the estimated requirement, the rate of improvement decreased as muscle growth approaches the genetically predetermined capacity despite sufficient nutrient supply (Eleroğlu et al., 2014; Reis et al., 2023). The decrease in percentage of liver weight with increasing dietary Arg levels, together with the lack of effects on kidney weight and abdominal fat, may be attributed genetic selection prioritizing muscle growth over visceral organ growth during overall body development (Kanlisi et al., 2024).

The LBL and QBL models for breast meat and carcass production were not significant. The non-linear regression model is an appropriate tool in modeling the biological responses where increasing levels of a particular nutrient alter the response (increase or decrease according to the parameter measured) up to a specific point (minimum or maximum requirement), after which the response plateaus (Alhotan et al., 2017). Therefore, when only a linear response is observed, or when a quadratic response shows a decrease in performance after the requirement is met, the data may not be suitable for an asymptotic broken-line model. The carcass weight and breast meat weight data response did not show a plateau due to the purely quadratic response. Furthermore, the linear response of the carcass yield data indicated the absence of a breakpoint and invalid model despite the significant P-value, which ultimately caused the Hessian matrix to be singular. On the other hand, the breast meat yield showed curvilinear responses, therefore, the dataset was fitted, and the models showed a tendency toward significance with relatively high R² values, suggesting a reasonable fit to the data, similar to those observed for growth performance. In addition, the requirements for breast meat yield from LBL and QBL models were 110.3% and 118.4%, respectively. These results are consistent with those of growth performance in the present study and previous research (Corzo et al., 2021). For a more precise estimation of Arg requirements for carcass production and breast meat production, using additional dietary Arg level may be recommended to ensure the detection of a distinct plateau response.

The dietary Arg level influenced breast meat quality, but its effects were not consistent. The supplementation of Arg improved breast meat pH by shifting it toward the normal range, decreasing from 6.31 to 5.89. Normal breast meat pH has ranged from 5.8 to 6.0 (Qiao et al., 2001; Van Laack et al., 2000). However, the dietary Arg level linearly increased drip loss, and this may be attributed to changes in muscle pH. The muscle pH, which directly influences protein denaturation, meat color, and drip loss, is a critical determinant of meat quality. The lower muscle pH could cause a decline in water holding capacity, increasing drip loss due to protein denaturation (Bowker and Zhuang, 2015; Qiao et al., 2001). In addition, the lower muscle pH may increase the reflectivity of myofibrillar proteins, and result in the loss of myoglobin, a pigment responsible for red meat color, from the muscle (Hui et al., 2001). These pH results were well associated with meat color and drip loss in the present study. However, Zampiga et al. (2018) have reported that Arg supplementation did not affect breast meat quality, which is inconsistent with the current findings. This discrepancy highlights the need for further research.

In the present study, the increase in SID Arg:Lys ratio did not influence the responses of Arg metabolism but resulted in increased BUN concentration. In broilers, Arg is catabolized into urea and ornithine via the arginase (Khajali and Wideman, 2010). Although arginase activity in the liver and kidney was not influenced by the dietary SID Arg levels in this study, the linear increase in BUN observed with greater SID Arg levels may be explained by a greater supply of substrate for urea and ornithine synthesis. This increase likely reflects an increased requirement for ornithine that are associated with cell proliferation and development (Castro and Kim, 2020; Liu and Kim, 2023), which coincided with the improved growth performance observed in the current study. Traditionally, BUN has been considered as an indirect indicator of protein metabolism and utilization in broilers (Donsbough et al., 2010; Ismoyowati et al., 2022). However, this interpretation is complicated by the fact that broilers excrete nitrogenous waste primarily through the uricotelic pathway. Previous research has suggested that uric acid is a more appropriate indicator of protein utilization than BUN (Bideshki et al., 2023). Therefore, the BUN concentration may serve as a more suitable indicator of Arg utilization rather than overall protein utilization. However, as ornithine concentrations were not analyzed in the present study, further research should include measurements of ornithine to clarify its role in Arg metabolism.

Furthermore, Arg is also catabolized to NO and citrulline through NOS in broilers, and NO production is stimulated under immunological and thermal stress, which can increase Arg requirements (Khajali and Wideman, 2010; Uyanga et al., 2022). The activity of NOS in the liver and kidney was not affected by the SID Arg:Lys ratio, like arginase, indicating that Arg requirement may not be influenced. Nevertheless, in the current study, the NO concentration in liver and kidney were not affected by SID Arg:Lys ratio levels, unlike BUN. This discrepancy may be attributed to the fact that the half-life of nitrate and nitrite may be short enough to detect differences in NO production. The half-lives of nitrate and nitrite are usually less than one hour (Gehle, 2013; Lapidge and Eason, 2010), however, the studies on half-life of NO for broilers are limited, warranting further research on pharmacokinetics of NO metabolites in broilers. Given that Arg supplementation improved muscle production and growth performance and the metabolic responses, Arg supply appears to have been sufficient. Arg was not additionally consumed under stress-related metabolic conditions. Therefore, the Arg requirement estimate may primarily reflect Arg sufficiency rather than additional metabolic demand.

The increase of SID Arg:Lys ratio decreased linearly mRNA expression of ASS in liver. Broilers are uricotelic animals, characterized by limited activity of the ornithine transcarbamylase in the urea cycle, which converts ornithine into citrulline (Scope and Schwendenwein, 2020). Furthermore, the carbamoyl phosphate synthase involved in synthesizing citrulline from ammonia is also restricted (Fathima et al., 2023; Ma et al., 2025). These enzymatic limitations suggest that only citrulline, primarily produced from Arg via NOS, can be resynthesized into arginine via ASS and ASL (Fernandes and Murakami, 2010). Although ASS in the Arg synthesis pathway is the rate-limiting enzyme in the Arg synthesis pathway (Hao et al., 2004), the linear decrease of mRNA expression of ASS may not influence Arg requirement (Castro and Kim, 2020) because the activity of ASS in poultry is low to synthesize Arg de novo to a limited extent. Notably, since ASS activity, protein expression level, and concentration of its substrate were not analyzed, future studies evaluating the effects of Arg levels on Arg metabolism and synthesis should include analysis of these factors.

In conclusion, increasing the dietary SID Arg concentration improved growth performance, and carcass yield. Although BUN concentrations and ASS mRNA expression were influenced, overall Arg metabolism was not altered by increasing dietary SID Arg levels to an extent that would impact SID Arg requirements. The estimated SID Arg:Lys ratio requirements based on LBL model for BWG were generally consistent with current Arg recommendations, however, the higher requirements estimated using the QBL model, together with the continued improvements observed in growth performance, carcass yield, and breast meat quality at elevated dietary Arg levels, indicate that supplying SID Arg above conventional recommendations may be beneficial for optimizing feed efficiency and carcass characteristics.

The SID Arg:Lys ratio requirements of male broiler from 10 to 21 days were estimated to be 116.4% and 123.4% for G:F based on LBL and QBL models, respectively. The estimated SID Arg:Lys ratio requirements of male broiler from 21 to 31 days were 105.6% for BWG using LBL model and 108.1% and 117.3% for G:F based on LBL and QBL models, respectively. The SID Arg:Lys ratio requirements of male broiler from 10 to 31 days were estimated to be 106.7% and 116.4% for BWG and 109.4% and 119.6% for G:F based on LBL and QBL models, respectively.

While the estimated SID Arg:Lys requirements for BWG remained relatively constant across the experimental periods, age-related variation was observed in G:F-based estimates, particularly when estimated via the QBL model. These findings highlight the importance of both age and response criteria in determining optimal Arg:Lys ratios and suggest that requirement estimates may differ depending on the production objective. Therefore, further research is warranted to clarify the mechanisms underlying these differences and to refine Arg requirement estimates under varying physiological and production conditions.

## CRediT authorship contribution statement

**Inho Cho:** Writing – original draft, Visualization, Validation, Software, Methodology, Investigation, Formal analysis, Data curation. **Jisoo Tak:** Writing – review & editing, Resources, Conceptualization. **Changsu Kong:** Writing – review & editing, Visualization, Validation, Supervision, Software, Resources, Project administration, Methodology, Investigation, Funding acquisition, Data curation, Conceptualization.

## Disclosures

The authors declare that they have no known competing financial interests or personal relationships that could have appeared to influence the work reported in this paper.
